# The General Medical Council

**Published:** 1894-05-26

**Authors:** 


### The Hospital

An Institutional Journal of

The Medical Sciences and Hospital Administration, with

Vol. XVI.—No. 400.] AN EXTRA NURSING SUPPLEMENT. [May 26, 1894.

### The General Medical Council.

The General Medical Council commenced its first session for the year 1894 on Tuesday last; and, upon its rising, we purpose to lay before our readers a summary of its proceedings. In the meanwhile, however, as the Council has often been subjected to criticism based upon misconception of its powers and duties, it may not be without interest to consider what it ought to accomplish, and to what extent it may be held to have fulfilled the objects for which it was established by the Legislature. These objects were two in number; the first being to secure that no person should be placed upon the Medical Register, as a legally qualified practitioner, until he had passed a proper and sufficient examination; and the second being to secure that no person should be suffered to remain upon the register if he had been guilty of legal criminality or of serious professional misconduct. The Council was a council of education and registration, and nothing more; and it was constituted by the Medical Act of 1858. Prior to the passing of that Act, qualifications or licences to practice were only valid in the division of the United Kingdom in which they had been obtained; that is to say, English qualifications only in England, Scottish only in Scotland, and Irish only in Ireland. In each division the qualifying corporations or universities were comparatively few in number, and the relative values of their several diplomas or licences were approximately known to the general public and to patients. There was no probability of confusion between a doctor of medicine of Oxford or Cambridge and a member of the College of Surgeons; while, in England, a qualification conferring the title of doctor was only taken by men who aspired to the higher branches of consulting practice, so that it actually represented, and was intended to represent, an education, both general and professional, of a very superior kind. The Act of 1858 abolished the distinctions between the divisions of the kingdom, and extended to every qualifying body the privilege of giving a license which should be valid in all. The qualifying bodies were nineteen in number, and some of them were in the habit of conferring the doctorate after an education very inferior to that which it usually represented in England. It was feared that the nineteen bodies, more or less competing for the fees paid by students for licenses, might in time be induced to lower their respective standards of examination; more especially in the cases of candidates who came from a different division of the kingdom, and were suspected of an intention to return to it; and that the bodies might thus increase their funds by attracting the very people whom it was most desirable, in the true interests of the public, to repel. In order to provide against the anticipated evil, the Medical Council was called into existence by the Act, and was authorised to exercise such a control over examinations as might lead to uniformity of requirement. The powers conferred upon the Council by the Act were those of visiting examinations, of making recommendations to the bodies responsible for them, and of presenting to the Privy Council any of these bodies whose examinations, in the opinion of the Council, fell short of reasonable and proper requirements. Power was also given to purify the Register by removing from it the name of any practitioner who had been convicted of any felony or misdemeanour, or who, in the judgment of the Council itself, had been guilty of "infamous conduct in a professional respect."

As modified in constitution by Acts subsequent to that of 1858, and intended to extend or to alter its provisions, the Council now consists of thirty persons, of whom six are appointed by the Crown, presumably as guardians of the interests of the public; five are elected by the votes of medical practitioners, and are commonly called "direct representatives"; while the remaining nineteen are appointed by as many licensing bodies. The business of the Council is what it always was, namely, to supervise education and examination, and to hear complaints against registered practitioners. In the discharge of the former duty it has appointed an inspector, who, together with a "visitor," who must himself be a member of the Council, in turn inspects and reports upon every examination in the United Kingdom by which a licence to practise is conferred, and renders a minute account of its character and of the methods by which it is conducted. Acting upon the information thus obtained, and after due consideration of any replies received from the examining bodies concerned, the Council has from time to time issued "recommendations," which, although they have no legal force, have yet, in almost every instance, been accepted and acted upon, with the general result that the period of study and the standard of proficiency required for a qualifying medical examination is everywhere practically the same. The English student has no temptation to resort to Scotland or to Ireland; and the Scottish or Irish students have no temptation to come to England. Trifling differences as to fees are questions which concern the examining bodies alone, the functions of the Council being limited to securing a practical uniformity of curriculum, and an uniform standard of examination, while, at the same time, it has fallen within their functions to extend the curriculum and to increase the requirement, in harmony with the ever-increasing extension and development of medical science. The last great step taken by the Council has been to increase the period of professional study from four years to five; and the natural corollary of this increase will be that more will be demanded from the students of the future, whose professional education has been spread over five years, than it would have been right to demand from those who had been admitted to examination after four years only. Besides the final, the preliminary and the intermediate examinations have received much attention from the Council, and the best commentary upon their labours is furnished by the fact that, until last year, no occasion ever arose for suggesting to an examining body the very possibility of presentment to the Privy Council. The members of the Medical Council, with very few exceptions, are themselves old and experienced teachers in schools of medicine; and they are perfectly well able to distinguish between practicable and impracticable requirements. The relations borne by the majority of them to examining bodies have also proved to be a source of strength to the Council as a whole, by providing that every such body shall have a voice in determining the representations which are made to it, and by removing any strain which might be produced by a demand for submission to any external authority.

In the way of purifying the Register, the Council, every session, is called upon to hear complaints that are made against registered practitioners; and, if these complaints are duly proved, and are of a sufficiently serious character, the Council removes the name of the offender from the Register, and deprives him of the privileges which registration affords. He can no longer recover charges for "professional attendance," he cannot hold any public medical appointment, he cannot give a valid "medical" certificate, and he cannot give medical evidence in a court of law. He is no longer a member of the medical profession, and the Council ceases to have any power over him. Being, in regard to cases of this class, a judicial body, the Council cannot be a prosecuting body also, but must wait until charges are made against the supposed culprit. Moreover, the Council can only decide upon the evidence submitted to it, and some cases have broken down on account of the imperfect manner in which they have been prepared by the complainants. On the other hand, many of the decisions of the Council have been appealed against in courts of law, and in every instance they have been upheld. The only exception, and that an apparent one, was furnished by the case of a dentist, against whom the Council had proceeded under the provisions of the Medical Act instead of under those of the Dentists Act, and had thus made a technical slip in the manner of discharging its duty. The name which had been removed was ordered to be restored to the Register, the Judge at the same time suggesting that the Council might take fresh action under the proper statute, and remove it again, a course which was forthwith pursued. The complaint most commonly brought against the Council, that it has done nothing for the suppression of "illegal" practice, is at once perfectly true and perfectly frivolous. The Council has no power to interfere with any person who is not a registered medical practitioner, and, in England, there is no such thing as "illegal" practice. Anybody, or everybody, is entitled to practise medicine or surgery, provided that he does not assume any title implying the possession of a legal qualification. Anybody may employ a carpenter, if he so pleases, to perform an amputation, or a shoemaker to treat a case of enteric fever, and either tradesman would be entitled to charge for labour and materials. Either would be liable to pay damages for any injury sustained by the patient which was traceable to "want of reasonable skill or care" on the part of the person employed. The country druggist or the itinerant quack stands in the same position as the carpenter or the shoemaker, and the Medical Council has not a shadow of authority over any of them. Its powers are strictly limited to registered members of the medical and dental professions; and, with regard to these, the policy which has been adopted has been to give warning that such or such conduct would for the future be considered "infamous in a professional respect," before proceeding to inflict the full penalty upon offenders. In the coming session, for example, it will be considered whether the advertisements issued by certain dentists are not sufficiently fraudulent and mendacious to be made the subjects of warning, and afterwards, if the warning should be ineffectual, of adjudication. The only occasion on which the Council has been able to act as a prosecuting body are a few in which men have incurred a penalty by falsely pretending to be medical practitioners when they were not so, and in these cases, as guardians of the register, the Council has taken proceedings before magistrates, and has sustained convictions against appeal. None can be more conscious than the members of the Council themselves of the frequent inadequacy of the powers entrusted to them; none would be more willing that these powers should be extended. But in the present position of public business, and in an intellectual atmosphere favourable to the encouragement of roguery, and unwilling to protect fools from the consequences of their folly, there is very small reason to expect that additional powers will be conferred. In the meanwhile, and until such powers are conferred, the Council should be judged by the use which it has made of the authority which it possesses, and not, as has been done by many of its critics, by the use which it has not made of the authority which it does not possess.

